# Comparison of platelet‐rich plasma efficacy with and without photoactivation in melasma: A randomized double‐blind study

**DOI:** 10.1111/jocd.16540

**Published:** 2024-08-23

**Authors:** Filiz Topaloğlu Demir, Ece Altun

**Affiliations:** ^1^ Faculty of Medicine, Department of Dermatological and Venereal Diseases Istanbul Medipol University Istanbul Turkey

**Keywords:** melasma, photoactivation, platelet‐rich plasma

## Abstract

**Background:**

Photoactivation has been suggested to enhance the efficacy of platelet‐rich plasma (PRP) in conditions other than dermatological diseases.

**Aims:**

To evaluate the efficacy of photoactivated PRP (P‐PRP) treatment for melasma by comparing it with non‐photoactivated, classical PRP (C‐PRP).

**Methods:**

The study consisted of 38 female patients diagnosed with melasma between April 2022 and May 2023. The patients were randomized into the P‐PRP and C‐PRP groups. Three sessions of P‐PRP or C‐PRP were applied to the patients at 2‐week intervals. The Melasma Area and Severity Index (MASI) and Melasma Quality of Life Index (MELASQoL) scores were compared before and 2 weeks after treatment.

**Results:**

The median age was 38 years, and the median disease duration was 60 months. Clinically, 94.7% of the cases were centrofacial and 5.3% were malar. According to Wood's lamp examination, 55.3% of the cases were epidermal, 13.2% were dermal, and 31.6% were mixed‐type. The median pre‐ and post‐treatment scores were 14.5 and 9, respectively, for MASI and 36.5 and 17, respectively, for MELASQoL. The post‐treatment MASI and MELASQoL scores of both groups significantly decreased (*p* < 0.001 for both). However, the intergroup difference was not significant. When all patients were evaluated together a moderate, positive, and significant relationship was detected between PRP and the pre‐ and post‐treatment MASI and MELASQoL scores (*r* = 0.494 and *p* = 0.002). No side effects associated with PRP were observed.

**Conclusion:**

PRP is an effective and safe treatment method for melasma. Further studies are needed to evaluate the contribution of photoactivation to PRP treatment in melasma.

## 1 INTRODUCTION

Melasma is a common, chronic, recurrent pigment disease with a pathogenesis that has not yet been fully elucidated, and it has negative effects on the quality of life in cosmetic terms.^1^, ^2^ Although there is not yet a curative treatment option for melasma, various topical and systemic treatments, as well as various procedures such as peeling, microneedling, laser, and light systems, are used to prevent and control the disease.^3^ Platelet‐rich plasma (PRP) treatment has recently emerged as a novel treatment option for melasma.^2^ The efficacy of this treatment has been attributed to multiple growth factors that reduce melanogenesis through various signal transduction pathways.^1^ Photoactivation has been suggested to enhance the efficacy of PRP by facilitating the release of growth factor from platelets in conditions other than dermatological diseases.^4^, ^5^ Although there are publications in the literature indicating that PRP treatment is effective in the treatment of melasma, the efficacy of photoactivated PRP (P‐PRP) has not been investigated in this condition.^1^, ^2^ In the current study, the efficacy of P‐PRP in the treatment of melasma was evaluated by comparing it with non‐photoactivated, classical PRP (C‐PRP).

## 2 MATERIALS AND METHODS

The study included 38 female patients aged 18 and over who presented to our outpatient clinic between April 1, 2022, and May 15, 2023 and were diagnosed with melasma. Patients with an active systemic disease, those using systemic medications, those using oral contraceptives, and pregnant and lactating women were excluded from the study. Only patients who were able to shield themselves from the sun and apply sunscreen during the treatment were included in the study. The patients were randomized into two groups by estheticians to receive P‐PRP or C‐PRP treatment, based on the order of their enrollment in the study. The T‐lab‐PRP kit was used to obtain PRP. The obtained PRP was exposed to activation radiation for 9 min at a wavelength of 645 nm and a 60‐degree angle from a distance of 3.5 cm. The GCELL® Autologous Tissue Suspension Technology was used for photoactivation.

The patients received a total of three sessions of intradermal P‐PRP or C‐PRP, depending on their groups, on the entire face with an interval of 0.5–1.0 cm every 2 weeks. All patients receiving P‐PRP or C‐PRP treatment were evaluated in terms of age, disease duration, age at disease onset, smoking or alcohol habits, pregnancy history, number of pregnancies, the presence of melasma before pregnancy, previous treatment history, sunscreen use, family history, the Fitzpatrick skin type, clinical type, melasma type according to Wood's lamp examination, and the Melasma Area and Severity Index (MASI) and Melasma Quality of Life Index (MELASQoL) scores before and after treatment (sixth week). The post‐treatment MASI and MELASQoL scores and the percentage improvement in these scores were compared for the delta (Δ) MASI and delta (Δ) MELASQoL scores. The study was approved by the local ethics committee of the affiliated university (number: E‐10840098‐772.02‐287/14.01.2022).

### 2.1 Statistical analysis

To summarize the data obtained from the study, descriptive statistics for continuous (numerical) variables were presented as median, minimum, and maximum values based on their distribution. Categorical variables were described in terms of numbers and percentages. The normality of the data distribution for numerical variables was assessed using the Shapiro–Wilk, Kolmogorov–Smirnov, and Anderson‐Darling tests. For comparisons between categorical variables based on treatment options, the Pearson chi‐square test was employed for 2 × 2 tables with expected counts of 5 or more. For tables with expected counts below 5, Fisher's exact test was utilized, and for RxC tables with expected counts below 5, the Fisher–Freeman–Halton test was applied. In cases where numerical variables did not follow a normal distribution for independent two‐group comparisons, the Mann–Whitney U test was used.

To calculate the percentage change in the MASI score, we subtracted the pre‐treatment MASI score from the post‐treatment score, divided this difference by the pre‐treatment MASI score, and then multiplied the result by 100. These percentage change values were utilized to assess the efficacy of each treatment. Spearman's rho correlation coefficient was employed to assess the relationship between the delta MASI and delta MELASQoL scores. For statistical comparisons between the pre‐ and post‐treatment MASI and MELASQoL scores, the Wilcoxon test was employed when numerical variables were not normally distributed.

Statistical analyses were conducted using the Jamovi (Version 2.3.28) and JASP (Version 0.17.3) software. A *p*‐value of 0.05 was deemed significant in these analyses.

## 3 RESULTS

The study included a total of 38 patients, of whom 19 underwent C‐PRP treatment and 19 received P‐PRP treatment. The median age of the participants was 38 years. The median age at disease onset was 32.5 years, and the median disease duration was 60 months. Clinically, 36 (94.7%) of the patients were diagnosed with the centrofacial type of melasma, and two (5.3%) with the malar type. According to the Wood's lamp examination, 21 (55.3%) patients had epidermal melasma, five (13.2%) had dermal melasma, and 12 (31.6%) had a mixed type. The clinical and demographic characteristics of the patients are summarized in Table 1.

**TABLE 1:** Descriptive statistics on demographic and clinical variables and comparisons between groups according to treatment type in patients with melasma.

	Overall	Treatment group		*p*
		C‐PRP (*n* = 19)	P‐PRP (*n* = 19)	
Age^b^	38.0 (22.0–51.0)	38.0 (25.0–51.0)	38.0 (22.0–47.0)	0.474^c^
Disease duration (months)^b^	60.0 (4.0–240.0)	72.0 (12.0–240.0)	48.0 (4.0–240.0)	0.421^c^
Age at disease onset^b^	32.5 (15.0–45.0)	29.0 (20.0–40.0)	33.0 (15.0–45.0)	0.279^c^
Smoking^a^, *present*	9 (23.7)	4 (21.1)	5 (26.3)	0.999^d^
Alcohol use^a^, *present*	3 (7.9)	1 (5.3)	2 (10.5)	0.999^d^
Pregnancy history^a^, *present*	23 (60.5)	10 (52.6)	13 (68.4)	0.507^d^
Number of pregnancies^b^	2.0 (1.0–4.0)	2.0 (1.0–2.0)	2.0 (1.0–4.0)	0.533^c^
Was melasma present before pregnancy?^a^ *yes*	17 (73.9)	8 (80.0)	9 (69.2)	0.660^d^
Do melasma symptoms increase in pregnancy?^a^ *yes*	7 (30.4)	2 (20.0)	5 (38.5)	0.405^d^
Is there a history of treatment for melasma?^a^ *yes*	12 (31.6)	5 (26.3)	7 (36.8)	0.727^d^
Sunscreen use^a^, *present*	32 (84.2)	16 (84.2)	16 (84.2)	0.999^d^
Sun exposure per day^a^				
<1 h	24 (63.2)	13 (68.4)	11 (57.9)	0.707^d^
1–2 h	8 (21.1)	3 (15.8)	5 (26.3)	
2–3 h	5 (13.2)	2 (10.5)	3 (15.8)	
>4 h	1 (2.6)	1 (5.3)	0 (0.0)	
OCS use^a^, *yes*	9 (23.7)	6 (31.6)	3 (15.8)	0.447^d^
Family history^a^, *present*	19 (50.0)	10 (52.6)	9 (47.4)	0.999^d^
Degree of closeness to the family member with a history of melasma^a^				
First degree	18 (94.7)	8 (88.9)	10 (100.0)	0.474^d^
Second degree	1 (5.3)	1 (11.1)	0 (0.0)	
Fitzpatrick skin type^a^				
3	19 (50.0)	12 (63.2)	7 (36.8)	0.194^d^
4	19 (50.0)	7 (36.8)	12 (63.2)	
Clinical type^a^				
Centrofacial	36 (94.7)	17 (89.5)	19 (100.0)	0.486^d^
Malar	2 (5.3)	2 (10.5)	0 (0.0)	
Melasma type (Wood's lamp examination)^a^				
Epidermal	21 (55.3)	12 (63.2)	9 (47.4)	0.523
Dermal	5 (13.2)	3 (15.8)	2 (10.5)	
Mixed	12 (31.6)	4 (21.1)	8 (42.1)	

Abbreviations: C‐PRP, classical platelet‐rich plasma; OCS, oral contraceptive; P‐PRP, photoactivated platelet‐rich plasma.

^a^ Number (percentage).

^b^ Median (minimum‐maximum).

^c^ Mann‐Whitney U test.

^d^ Pearson chi‐square test/Fisher's exact test/Fisher‐Freeman‐Halton test.

Comparisons based on treatment type revealed no significant differences in age, disease duration or onset, smoking or alcohol habits, pregnancy history, number of pregnancies, the presence of pre‐pregnancy melasma, symptom exacerbation during pregnancy, prior treatments, sunscreen use, daily sun exposure duration, oral contraceptive use, family history, degree of closeness to the family member with a melasma diagnosis, the Fitzpatrick skin type, clinical type, or melasma type (*p* > 0.05 for all) (Table 1).

The median pre‐treatment MASI score was 14.5, which decreased to 9 after treatment. For the MELASQoL scores, the median value was 36.5 before treatment and 17 after treatment. No significant difference was observed between the treatment groups in terms of pre‐ or post‐treatment MASI scores (*p* = 0.815 and *p* = 0.455, respectively). However, both treatment groups demonstrated a significant reduction in the post‐treatment MASI scores (*p* < 0.001 for both). Similarly, no significant difference was found between the treatment groups for the pre‐ or post‐treatment MELASQoL scores (*p* = 0.373 and *p* = 0.726, respectively). Yet, both groups showed a significant reduction in the post‐treatment MELASQoL scores (*p* < 0.001 for both) (Table 2).

**TABLE 2:** Intragroup and intergroup comparisons of symptom severity and quality of life in patients with melasma before and after treatment.

	Overall	Treatment group		*p* ^a^
		C‐PRP (*n* = 19)	P‐PRP (*n* = 19)	
Pre‐treatment MASI score	14.5 (4.0–27.0)	15.0 (4.0–24.0)	14.0 (6.0–27.0)	0.815
Post‐treatment MASI score	9.0 (0.0–20.0)	9.0 (0.0–20.0)	8.0 (4.0–14.0)	0.455
*p* ^b^	***<0.001***	***<0.001***	***<0.001***	
Pre‐treatment MELASQoL score	36.5 (20.0–70.0)	35.0 (21.0–70.0)	42.0 (20.0–70.0)	0.373
Post‐treatment MELASQoL score	17.0 (7.0–49.0)	17.0 (10.0–49.0)	16.0 (7.0–36.0)	0.726
*p* ^b^	***<0.001***	***<0.001***	***<0.001***	

*Note*: Data presented as median (minimum‐maximum).

Abbreviations: C‐PRP, classical platelet‐rich plasma; MASI, Melasma Area and Severity Index; MELASQoL, Melasma Quality of Life Index; P‐PRP, photoactivated platelet‐rich plasma.

^a^ Mann–Whitney U test.

^b^ Wilcoxon test.

No significant differences were found in treatment responses across the three types of melasma (epidermal, dermal, and mixed) (*p* = 0.874). Likewise, no significant differences were found between epidermal melasma and dermal or mixed melasma in terms of treatment response (*p* = 0.727). The improvement rates were similar among the patient groups categorized by varying degrees of MASI score improvement (0%–25%, 25%–50%, and 50% or higher). This was true across all types of melasma and even when melasma was classified into two categories (epidermal and dermal + mixed) (*p* = 0.211 and *p* = 0.625, respectively) (Table 3).

**TABLE 3:** Comparison of treatment response according to melasma type.

	Δ MASI score category			*p*
	0–25% (*n* = 12)	25–50% (n = 19)	≥50% (*n* = 7)	
Melasma type (wood's lamp examination)				
Epidermal	8 (66.7)	9 (47.4)	4 (57.1)	0.211
Dermal	3 (25.0)	2 (10.5)	0 (0.0)	
Mixed	1 (8.3)	8 (42.1)	3 (42.9)	
Melasma type (wood's lamp examination)				
Epidermal	8 (66.7)	9 (47.4)	4 (57.1)	0.625
Dermal + mixed	4 (33.3)	10 (52.6)	3 (42.9)	

*Note*: Fisher's exact test/Fisher–Freeman–Halton test.

Abbreviation: MASI, Melasma Area and Severity Index.

In considering improvement as two categories (less than 50% improvement [suboptimal improvement] vs. 50% or more improvement [adequate improvement]), no significant difference was found in patients treated with C‐PRP or P‐PRP (*p* = 0.999). Similarly, when considered in three categories based on the MASI score improvement rates (0%–25%, 25%–50%, and 50% or higher), neither C‐PRP nor P‐PRP treatments had a significant impact on improvement (*p* = 0.153). However, the number of patients showing a decrease of 25%–50% in the post‐treatment MASI scores compared to pre‐treatment scores was 1.7 times higher in the group that received P‐PRP (Table 4).

**TABLE 4:** Comparison of the MASI score change percentages between the treatment groups.

	Treatment group		*p*
	C‐PRP (*n* = 19)	P‐PRP (*n* = 19)	
Δ MASI score category			
0%–25%	9 (47.4)	3 (15.8)	0.153
25%–50%	7 (36.8)	12 (63.2)	
≥50%	3 (15.8)	4 (21.1)	

*Note*: Pearson chi‐square test/Fisher–Freeman–Halton test.

Abbreviations: C‐PRP, classical platelet‐rich plasma; MASI, Melasma Area and Severity Index; P‐PRP, photoactivated platelet‐rich plasma.

**FIGURE 1:**
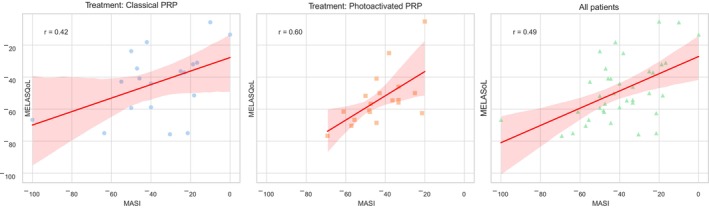
Correlation between Δ MASI and Δ MELASQoL.

Upon examining the entire sample, irrespective of treatment type, a moderate, positive, and significant relationship was found between an increase in the improvement of melasma severity following treatment and enhancement in quality of life (*r* = 0.494, *p* = 0.002) (Figure 1).

**FIGURE 2:**
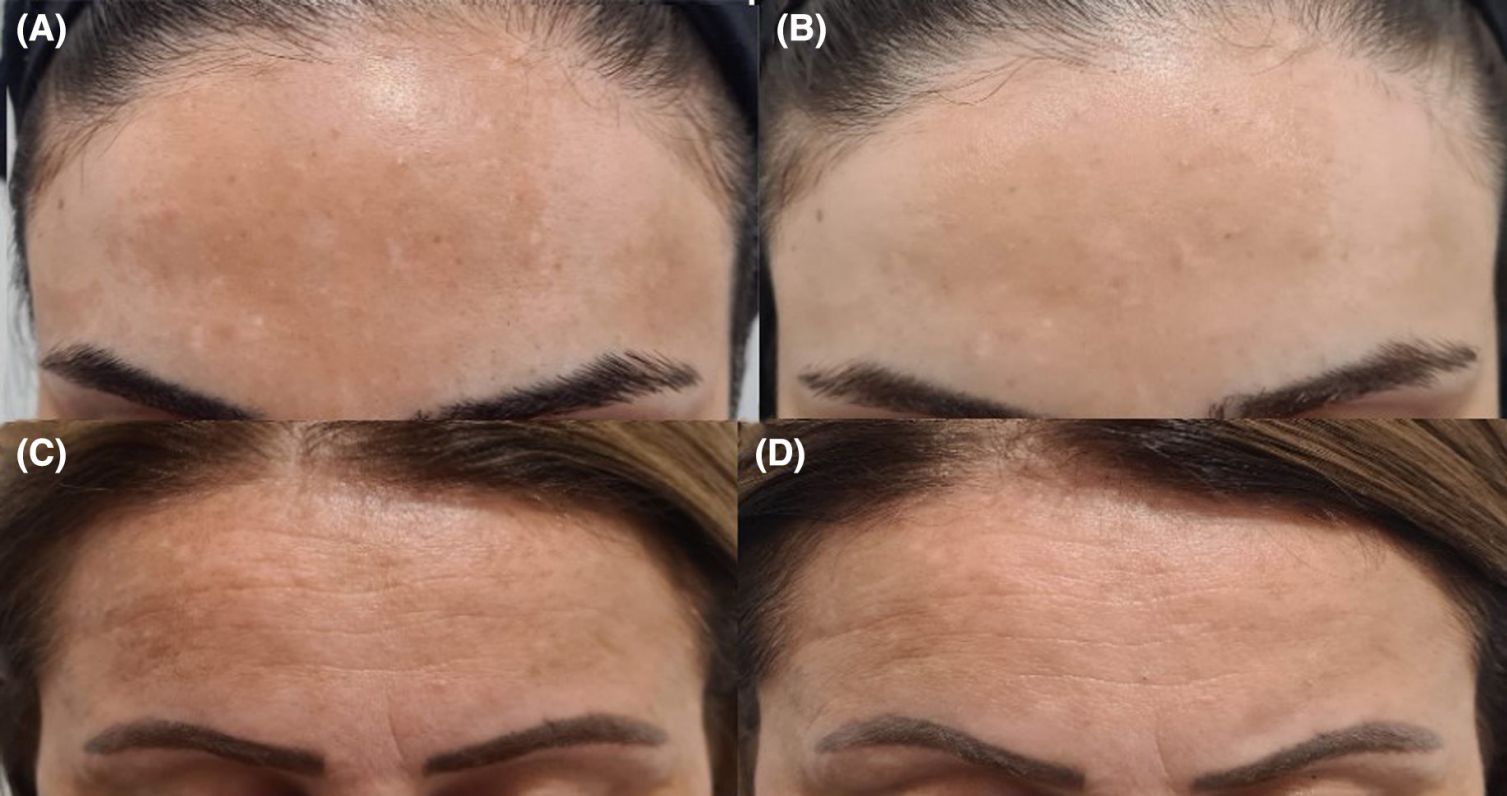
Melasma patient treated with C‐PRP, (A) before treatment, (B) after treatment. Melasma patient treated with P‐PRP, (C) before treatment (D) after treatment.

**FIGURE 3:**
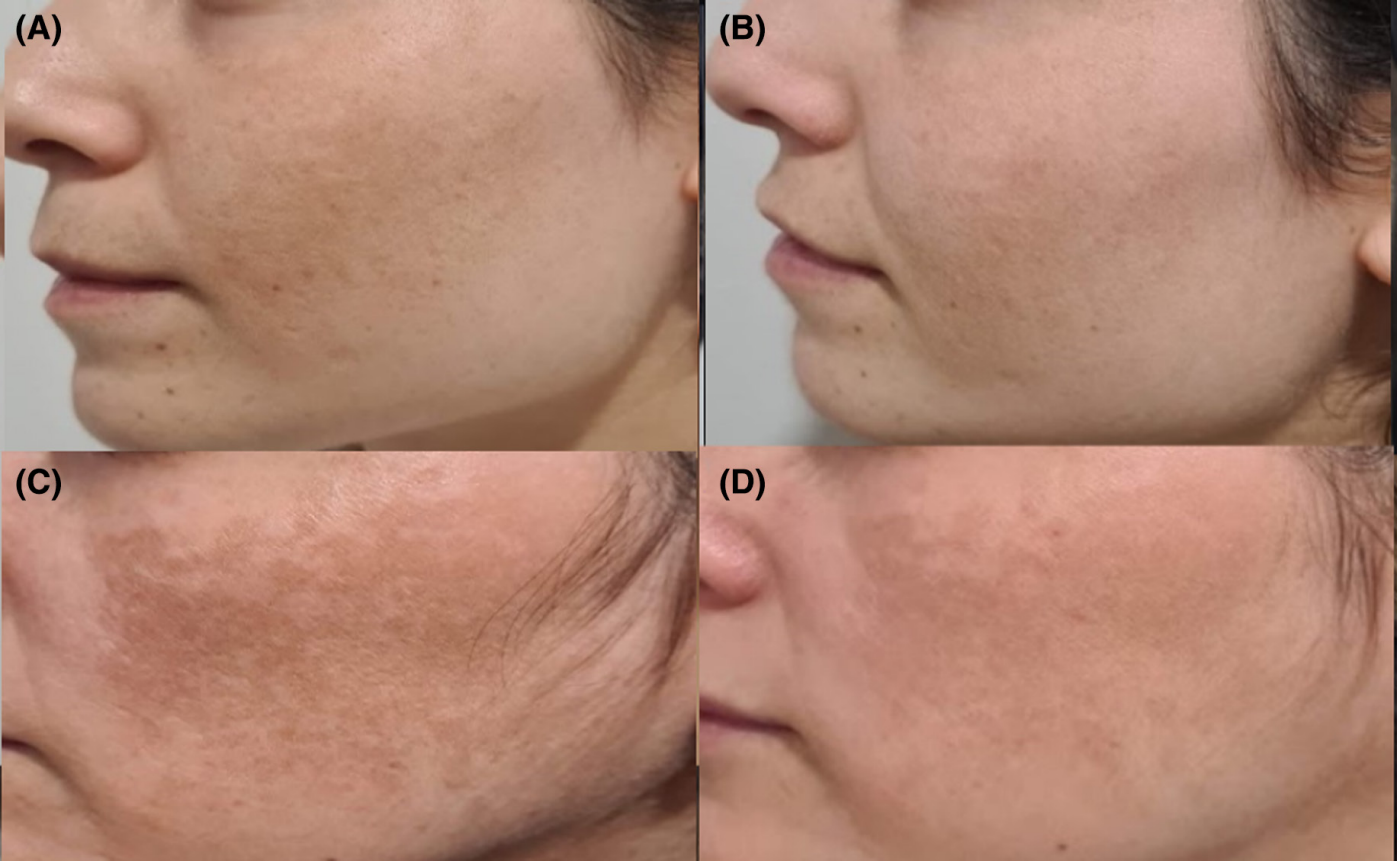
Melasma patient treated with C‐PRP, (A) before treatment, (B) after treatment. Melasma patient treated with P‐PRP, (C) before treatment (D) after treatment.

Patients treated with C‐PRP or P‐PRP were shown with before and after treatment photographs. (Figures 2 and 3).

## 4 DISCUSSION

PRP is an autologous preparation that contains a four‐ to seven‐times higher concentration of platelets and is rich in fibrinogen, fibrin, chemokines, and leukocytes.^6^, ^7^ It contains more than 30 growth factors, including platelets, vascular endothelial growth factor (VEGF), fibroblast growth factor (FGF), platelet‐derived growth factor (PDGF), transforming growth factor beta (TGF‐β) 1 and 2, epidermal growth factor (EGF), platelet‐derived angiogenesis factor, hepatocyte growth factor, and insulin‐like growth factor 1 and 2.^7^, ^8^ The efficacy of PRP has been attributed to these growth factors found in platelet alpha granules, which stimulate osteoblasts, adult mesenchymal stem cells, endothelial cells, fibroblasts, and epidermal cells.^8^, ^9^, ^10^

Although the etiology of melasma has not yet been fully understood, its pathogenesis has been attributed to the interaction between hormonal levels, UV light, inflammation, and free radical formation, affecting keratinocytes, fibroblasts, and, thus, melanogenesis.^11^ The efficacy of PRP in melasma has also been associated with growth factors, such as TGF‐β1, TGF‐β2, PDGF, and EGF, which reduce melanogenesis through various signal transduction pathways.^1^, ^6^ Tyrosinase is the rate‐limiting enzyme in melanogenesis and can be inhibited through an effect on transcription factors, such as microphthalmia‐associated transcription factor, as well as through delayed activation of extracellular signal‐associated kinase activation.^6^ TGF‐β1 suppresses melanogenesis by downregulating activity promoting microphthalmia‐associated transcription factor and inhibiting the expression of the paired box homeo‐c gene, which reduces the production of tyrosinase and tyrosinase‐related proteins 1 and 2 at the protein level.^9^, ^12^ EGF reduces melanin production by inhibiting prostaglandin E2 expression and tyrosinase enzyme activity.^13^ PDGF, found in PRP, provides “shine” to the skin by participating in blood vessel formation, collagen synthesis, and hyaluronic acid formation.^14^ It is considered that the curative effect of PRP in melasma is related not only to pigment metabolism but also to its multiple repair functions, antibacterial or anti‐inflammatory effects, and blood vessel restructuring function that cannot be detected in the skin.^2^, ^15^

PRP has shown significant improvement in melasma based on the moderate recommendation level.^16^ In a study evaluating 40 patients with melasma, Tuknayat et al.^1^ detected an average 54.5% decrease in the patients' modified MASI scores after a total of three sessions of intralesional PRP administration, with each session occurring once a month. A controlled study comparing intradermal PRP with intradermal normal saline injection showed significant improvement in MASI scores.^17^ A meta‐analysis evaluating 10 studies involving 395 adults reported that PRP, in combination with other therapies or alone, provided a significant reduction in modified MASI scores. In the overall effectiveness evaluation of PRP, it has been shown that patients or doctors are highly satisfied with the treatment of melasma with PRP.^2^ In our study, PRP treatment was evaluated to be effective in melasma, similar to the literature. Treatment efficacy was supported by the post‐treatment changes in the MASI scores compared to the pre‐treatment scores, as well as the change in the MELASQoL scores. Unlike the literature,^16^ reporting that the treatment response of patients with epidermal melasma is higher than that of patients with mixed‐type melasma, as indicated by 50% and 20.64% improvements in the MASI score, respectively, we observed no significant difference in treatment response according to melasma type.

In the literature, in order to increase the efficacy of PRP treatment, it has been combined with microneedling, light systems such as intense pulsed light, and topical treatments.^2^, ^16^ In the current study, instead of combined treatments, we utilized photoactivation to increase the efficacy of PRP treatment for melasma and enhance PRP treatment. PRP activation before injection is an important issue that needs to be discussed and improved. In the bloodstream, platelets exist in a resting, discoid state unless specifically activated by stimuli. Therefore, PRP needs to be activated to release growth factors from platelets faster.^1^, ^4^, ^8^ Traditional activation reagents, such as collagen type I, thrombin, and adenosine diphosphate, have certain disadvantages, including allergenic reactions and toxicity. Furthermore, platelets can only be activated once with these reagents.^4^ Photostimulation based on low‐level laser (or light) therapy (LLLT) has been used in basic scientific research as well as in a variety of medical treatments, such as wound healing and pain control.^3^ Platelet activation, which involves direct integrin alphaIIß3 activation, granule release, aggregation, and thrombus formation, has been attributed to the mobilization of calcium from intracellular storage to the plasma membrane.^4^ In vitro release studies have shown that PRP photoactivated with a polychromatic light source releases PDGF, basic FGF, and TGF‐ß for a significantly longer time and in higher amounts than PRP activated with CaCl_2_.^4^ Hoffman and Monroe found that LLLT increased platelet activity.^18^

In a preliminary clinical study, light was applied to PRP with a commercial device (Adi‐Light) for the treatment of osteoarthritis, and it was reported that the pain scores of patients decreased.^19^ In another study, Paterson et al.^5^ showed that P‐PRP improved self‐reported pain, symptoms, and lower extremity function and concluded that this could provide a safe and effective new treatment for knee osteoarthritis. However, there is only one publication regarding the use of P‐PRP in dermatology. In that study, Mercuri et al.^20^ reported that a patient with linear morphea in coup de sabre was successfully treated with Meta Cell Technology (MCT®) plasma therapy, a new photothermal activated PRP. Before its administration to the patient, PRP was transferred to a special 10 mL medical container (MCT Kit®, Barcelona, Spain), which was placed in a photothermal stimulator machine (MCT Unit®, Barcelona, Spain) providing a controlled temperature of 4°C and a red light source at a wavelength of 623.5 nm. This photothermal activation process, which lasts 15 min, causes the acceleration of light absorption, electron transfer reactions, and adenosine triphosphate production in the electron transport chain of mitochondrial platelets, increasing intracellular calcium concentration (Ca2+), intercellular calcium mobilization. Lastly, it has been suggested to increase the release of growth factors, such as VEGF, EGF, and basic FGF.^20^

There are also conflicting results concerning the effect of light on platelets.^21^ Rola et al.^22^ showed that LLLT had an antiaggregative effect on platelets. It has been reported that cellular responses in the presence of photostimulation may vary depending on the application parameters, and that parameters such as light dose, power output, wavelength, and intensity play a crucial role in determining the efficacy of photostimulation.^21^

The limitations of the study are the small number of patients and the short follow‐up period.

In conclusion, this is the first study to evaluate the efficacy of P‐PRP in the treatment of melasma. We observed that the PRP application with both methods was effective in the treatment of melasma. Although there was no statistically significant difference between the efficacy of P‐PRP and C‐PRP, the number of patients with a 25%–50% decrease in MASI scores after treatment with P‐PRP was found to be 1.7 times higher when compared to the C‐PRP group. To establish the efficacy of P‐PRP, there is a need for further studies with larger patient groups that evaluate the parameters necessary for optimal photostimulation. The P‐PRP application can be a novel and exciting area of research in the field of dermatology.

## CONFLICT OF INTEREST STATEMENT

There is no conflict of interest.

## ETHICS STATEMENT

The study was approved by the local ethics committee of the affiliated university (number: E‐10840098‐772.02‐287/14.01.2022).

## Data Availability

The data that support the findings of this study are available from the corresponding author upon reasonable request.
